# The relationship between psychiatric symtoms and childhood trauma

**DOI:** 10.1192/j.eurpsy.2023.1917

**Published:** 2023-07-19

**Authors:** M. G. Almeida, M. E. C. Virginio, J. R. A. Almeida

**Affiliations:** 1 PUC; 2 Uel, londrina, Brazil

## Abstract

**Introduction:**

Recent evidences suggest that situations of abandonment, neglect and abuse are risks factores for onset of psychopatology in adulthood. This association occus in that traumatic events in the early stages of development and may trigger severe and disabling psychiatric disorders in adults.

**Objectives:**

The presente study aimed to evaluate the associations between the occurence and severity of chidhood trauma and the development of psychiatric disorders in hospitalized teenagers of the Hospital Psychiatric Vida Londrina/Pr.

**Methods:**

The sample was consisted of 14 psychiatric patients, evaluated for severity of psychiatric symtoms by the Back Scale for Suicid Ideation (BSI); Beck Depression Inventory (BDI); Beck Anxiety Inventory (BAI); Back Hopelessness Scale (HAD) and the Barratt Impulsiveness Scale (BIS-11).

**Results:**

In the sample studie, 84% of the teenegers reports that they had some type of childhood trauma. The results indicate that traumas are associated mainly with the development of mood disorders and also with increasin severity of psychiatric symptoms, specially the depressive symptoms, hopelessness, suicidal ideation and impulsivity. Teenagers with emocional neglect, emocional abuse and physical neglect demonstrated having five times more risk of personality disorder. Individuals who suffered physical abuse; sexual abuse and physical neglect presentes at three times more risk of commiting suicid attemps.

**Conclusions:**

These data demonstrate the relevance of trauma childhood as a trigger for psychiatric symptoms.

**Disclosure of Interest:**

None Declared

